# Labeling male anorectal malformations: objective evaluation of radiologic imaging before surgery

**DOI:** 10.3389/fped.2023.1224620

**Published:** 2023-08-03

**Authors:** A. Morandi, F. Maestri, M. Ichino, M. A. Pavesi, F. Macchini, A. Di Cesare, E. Leva

**Affiliations:** ^1^ Department of Pediatric Surgery, Fondazione IRCCS Ca’ Granda Ospedale Maggiore Policlinico, Milan, Italy; ^2^ Pediatric Radiology Unit, Fondazione IRCCS Ca’ Granda Ospedale Maggiore Policlinico, Milan, Italy; ^3^ Department of Clinical Sciences and Community Health, University of Milan, Milan, Italy

**Keywords:** anorectal malformation, radiological assessment, pediatric radiology, anorectal malformation classification, anorectal malformation assessment

## Abstract

**Purpose:**

Prone cross-table lateral x-ray (CTLxR) and colostogram aid surgical planning for anorectal malformations (ARMs) without perineal fistulas. We suggest objective imaging tools to classify ARMs.

**Methods:**

Three observers prospectively evaluated CTLxR and colostograms of male ARM patients (2012–2022) without perineal fistulas. The level of the rectal pouch was estimated with pubococcygeal (PC) and ischiatic (I) lines. On CTLxR, we described the “pigeon sign”, defined as the rectal pouch ending with a beak-like image, suspicious for a rectourinary fistula. ARM was defined as rectobulbar when the rectal pouch was below the I line, rectoprostatic when between PC and I lines, and rectovesical when above the PC line. Concordance was assessed with Fleiss' kappa. Sensitivity, specificity, positive predictive value (PPV), and negative predictive value (NPV) of the “pigeon sign” were calculated.

**Results:**

Thirteen patients were included in this study. The interobserver agreement on CTLxR was 69.2% (*k* = 0.54) on pouch ending, 84.6% (*k* = 0.69) on the “pigeon sign”, and 76.9% (*k* = 0.69) on diagnosis; concordance between observers and intraoperative diagnosis was 66.6% (*k* = 0.56). The “pigeon sign” had 75% sensitivity, 100% specificity, 100% PPV, and 50% NPV. The interobserver agreement on colostograms was 84.6% (*k* = 0.77) on pouch ending and 89.7% (*k* = 0.86) on diagnosis; concordance between observers and intraoperative diagnosis was 92.3% (*k* = 0.90).

**Conclusion:**

PC and I lines and the “pigeon sign” are useful tools in examining CTLxR and colostograms. Adequate CTLxR interpretation may modify surgical strategy.

## Introduction

Anorectal malformations (ARMs) comprise a wide spectrum of rare anatomical defects of the genitourinary and anorectal tracts (1). ARMs are classified according to the Krickenbeck classification (Table 1) (2). For male patients, this classification includes, as major clinical groups, the following ARM types: perineal fistulas, rectourethral fistulas, including bulbar and prostatic fistulas, rectovesical fistulas, ARMs with no fistulas, and anal stenoses (2). Functional prognosis is influenced by the type of ARM, the quality of the sacrum, and the presence of spinal defects. Surgery is necessary not only to repair the anatomy but also to offer the best chance of gaining continence (1, 3, 4). Intraoperative damage to anatomical structures, such as the genitourinary tract, nerves, and muscles, may occur if the specific anatomy of the defect is not well clarified preoperatively and if the surgical approach is not adequately planned. In addition, postoperative complications such as dehiscence, scarring, stenosis, and prolapse may worsen the prognosis (5). Accurate preoperative assessment of the patient is pivotal to providing the best surgical correction.

**Table 1:** Krickenbeck classification for ARM in male patients (2).

Major clinical groups in males	Rare/regional variants in males
Perineal (cutaneous) fistula	Pouch colon
Rectourethral fistula • Rectobulbar • Rectoprostatic	Rectal atresia/stenosis
Rectovesical fistula	H-type fistula
No fistula	Others
Anal stenosis	

Traditionally, a male patient born with ARM, in the absence of an evident orifice as for perineal fistulas, is first studied with prone cross-table lateral x-ray (CTLxR) after 24–48 h of life. A three-step approach is used: the opening of a stoma is created, which will provide the opportunity to perform high-pressure distal colostogram prior to planning the anorectoplasty (4). This investigation gives additional information about the presence and level of rectourinary fistulas. However, discordance in interpreting CTLxR and distal colostograms is reported, even among expert pediatric colorectal surgeons (6). To reach an objective evaluation of radiological imaging to assess the ARM type, we evaluated the utility and the accuracy of the systematic use of specific anatomical landmarks and signs during these assessments.

## Materials and methods

We performed a prospective observational study on male patients with ARMs who received an anorectoplasty at our center in the time period from July 2012 to February 2022. Inclusion criteria were the absence of a visible orifice on the perineum or scrotum at birth, the use of a three-step approach for correction, and a CTLxR and high-pressure distal colostogram performed at our center along with available images in our PACS (Picture Archiving and Communication System). Female patients and patients with perineal fistulas or anal stenoses were excluded. The operative diagnosis was reported according to Krickenbeck classification (2) as an ARM with a bulbar, prostatic, or rectovesical fistula or an ARM type of imperforate anus with no fistula. All images were collected and stored.

At our center, CTLxR is usually performed in the first 2 days of life. The patient is positioned prone, with support under the hips. A radiopaque marker is positioned on the skin at the level of the muscle complex. The patient is left in this position for at least 3 min before performing the lateral x-ray. Ideally, the CTLxR should be centered over the greater trochanters, and the two femurs should be perfectly aligned (7). At our center, CTLxR are recorded in the Neonatal Intensive Care Unit with an FDR nano Fuji mobile imaging device (radiologic exposure: 53 kV and 25 mAs, class of dose I) (8).

When opening a colostomy for patients with ARM, we generally choose to create a divided colostomy on the descending colon-sigmoid passage with a distal mucous fistula. The mucous fistula is then used to perform high-pressure distal colostogram before surgery (9). At our center, a dedicated team of pediatric surgeons and pediatric radiologists is always present to perform colostogram. An 8-Fr Foley catheter is inserted in the mucous fistula, and the catheter balloon is inflated with water to secure the catheter, avoid retrograde leakage of contrast, and provide high pressure in the distal bowel to visualize urinary fistulas. A radiopaque marker is positioned on the skin at the level of the muscle complex. The patient is placed in a lateral position with the hips flexed and the femurs aligned. A water-soluble contrast is injected in the Foley catheter under direct radioscopic control. When the distal pouch is visualized, the contrast injection is continued to fully distend the distal colon and rectum. With adequate pressure, the distal pouch overcomes the strength of the pelvic muscles and assumes a round-shaped profile. In addition, the high pressure will open and highlight the presence of a possible urinary fistula (10). Once adequate information is gained in the lateral position, an additional radioscopic evaluation with the fully distended colon in the anteroposterior position is performed to evaluate the length of the distal colon that will be available for surgical reconstruction (9). At our center, colostograms are recorded with a Luminos dFR Max fluoroscopy machine in a pulsed mode (radiologic exposure: 2.5 min, dose area product 23.28 Gycm^2^, reference air karma 0.70 mGy, class of dose I) (8).

**Figure 1:**
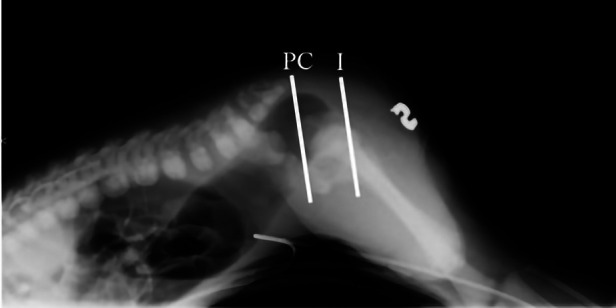
Pubococcygeal (PC) and ischiatic (I) lines are drawn. Level 1 is defined when the rectal pouch is above PC line, level 2 when between PC and I lines, and level 3 when below the I line.

**Figure 2:**
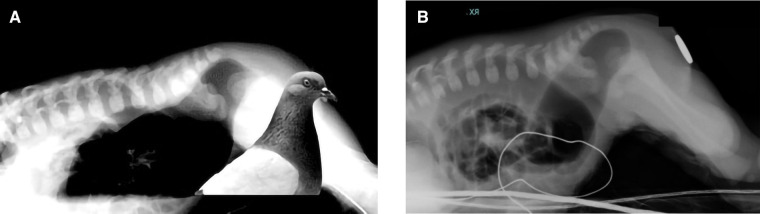
(**A,B**) “Pigeon sign” is highlighted. (**A**) Presence of the “pigeon sign”. (**B**) No “pigeon sign”.

**Figure 3:**
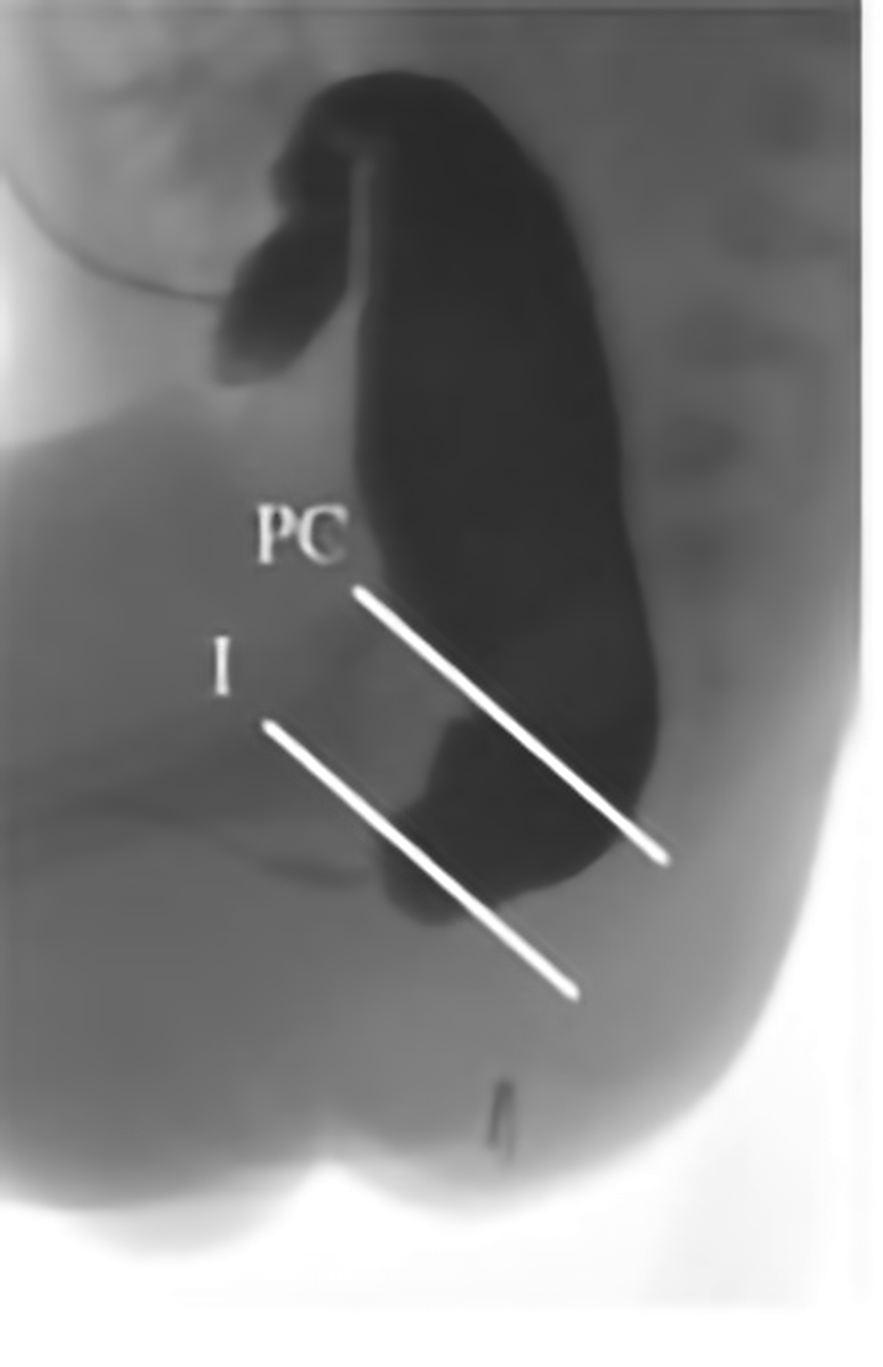
Pubococcygeal (PC) and ischiatic (I) lines are drawn. Level 1 is defined when the rectal pouch is above the PC line, level 2 when between PC and I lines, and level 3 when below the I line. This colostogram shows the presence of a fistula with the urinary tract below the I line, consistent with a rectobulbar fistula.

Images were blindly reviewed and scored by a pediatric surgeon consultant and two pediatric surgical trainees in pediatric surgery, all dedicated to colorectal surgery. To limit biases, images were randomly proposed to the observers, and the same image was randomly proposed twice to evaluate the objectiveness of the evaluation method. For CTLxR, the following landmarks were proposed: the pubococcygeal (PC) line, traced between the midpoint of the pubis and the inferior aspect of the lowest visible vertebra (11), and the ischiatic (I) line, traced parallel to the PC line and passing through the lowest visible ischiatic point (Figure 1). The level of the pouch was scored as 1 when ending above the PC line, 2 when between the PC and I line, and 3 when below the I line. Observers were also asked to evaluate on CTLxR the presence of the “pigeon sign”, defined as the rectal pouch ending with a triangular beak-like image, considered suspicious for a rectourinary fistula (Figure 2A). Figure 2B shows the radiologic aspect of the rectal pouch with no evidence of the “pigeon sign”. Based on the association between the level of the pouch and the presence/absence of the “pigeon sign”, the hypothesis of ARM type was formulated, as shown in Table 2. Concerning high-pressure distal colostogram, the same lines (PC and I) were adopted to define the level of the rectal pouch. The presence of the fistula was directly visualized by the contrast (Figure 3). ARM type was hypothesized based on the association between the level of the pouch (score 1–3) and the presence/absence of the fistula. Surgical reports were reviewed, and the definitive intraoperative diagnosis was recorded. All data were collected in a pseudonymized database and stored according to the Data Protection Act.

**Table 2:** Diagnostic hypothesis of ARM based on CTLxR findings.

Radiological signs at CTLxRx	Diagnostic hypothesis
No pigeon sign, levels 1–2–3	Imperforate anus with no fistula
Pigeon sign, level 1	Rectovesical fistula
Pigeon sign, level 2	Rectoprostatic fistula
Pigeon sign, level 3	Rectobulbar fistula

Agreement between the classifications of ARM at imaging and intraoperative diagnosis, as well as interobserver agreement were evaluated with Fleiss' Kappa. Intraobserver agreement for the images that were rated twice was also calculated. Results are expressed as free-level kappa (*k*) and the percentage of agreement. Interpretation of *k* is as follows: <0, poor agreement; 0.01–0.20, slight agreement; 0.21–0.40, fair agreement; 0.41–0.60, moderate agreement; 0.61–0.80, substantial agreement; and 0.81–1, almost perfect agreement (12). In addition, sensitivity, specificity, positive predictive value (PPV), and negative predictive value (NPV) of the “pigeon sign” were calculated on radiologic imaging when all observers agreed on its presence/absence.

## Results

In the study period, 114 patients with ARM received an anorectoplasty at our center. Of these, 50 (43.8%) patients were males. Of 50, 23 (46%) patients had an ARM type eligible for the study (ARM with no fistula or ARM with a urinary fistula). Twenty-one patients received the three-step approach. Of these patients, 13 (62%) received both CTLxR and high-pressure distal colostogram at our center and therefore met the inclusion criteria of our study. The remaining eight patients (38%) were outborn infants referred to our center after colostomy. Patients in the study had the following definitive intraoperative diagnosis: two ARMs with no fistula, nine with bulbar fistulae, one with a prostatic fistula, and one with a rectovesical fistula.

CTLxR was performed on average at 31.2 ± 12 h of life. Two CTLxR examinations were performed before the 24 h of life, specifically at 17 and 21 h of life. Concerning CTLxR, the interobserver agreement was moderate on the level of the rectal pouch (69.2%, *k* = 0.54), substantial on the presence of the “pigeon sign” (84.6%, *k* = 0.69), and substantial on hypothesized diagnosis, namely, the Krickenbeck ARM type (76.9%, *k* = 0.69). Concordance between observers and the final intraoperative diagnosis was moderate (66.6%, *k* = 0.56) on CTLxR. When excluding the two CTLxR examinations performed before 24 h of life, the interobserver agreement was moderate on the level of the rectal pouch (69.7%, *k* = 0.55), substantial on the presence of “pigeon sign” (81.8%, *k* = 0.64), and substantial on hypothesized diagnosis (78.7%, *k* = 0.72). Concordance between observers and the final intraoperative diagnosis was moderate (66.6%, *k* = 0.56). A total of 18 CTLxR images were randomly chosen and blindly evaluated twice by the same observer. The intraobserver agreement on the hypothesized diagnosis for subsequent evaluations was substantial (88.9%, *k* = 0.78). Ten CTLxR images were included to evaluate the sensitivity, specificity, PPV, and NPV of the “pigeon sign”. The “pigeon sign” resulted in having a sensitivity of 75%, a specificity of 100%, a PPV of 100%, and an NPV of 50%.

The mean age at colostogram was 68 ± 28.3 days. Considering colostograms, the interobserver agreement was substantial on the level of the rectal pouch (84.6%, *k* = 0.77) and almost perfect on hypothesized diagnosis (89.7%, *k* = 0.86). Concordance between observers and the final intraoperative diagnosis was almost perfect on colostograms (92.3%, *k* = 0.90). A total of 18 colostogram images were randomly chosen and blindly evaluated twice by the same observer. The intraobserver agreement for subsequent evaluations was perfect (100%, *k* = 1).

## Discussion

ARM represents a wide spectrum of defects and conditions. A clear understanding of normal anorectal anatomy and the different types of ARMs is necessary for planning surgery and its success. The type of ARM and other variables, such as sacral and spinal anomalies, also influence the functional outcome (1). A correct classification of the ARM is necessary to appropriately address the follow-up and management of possible complications. A uniform classification of ARM is also mandatory to make series comparable between centers (2, 6, 13).

ARM types have been historically presented with different classifications: the Melbourne classification in 1970, dividing the lesions into three groups (high, intermediate, and low); the Wingspread classification in 1984 (14), which was anatomically and embryologically oriented; the Peña classification (15), which abandoned the term “low,” “intermediate,” and “high” and proposed a classification based on the anatomical defects and how they correlate with surgical management; and the Krickenbeck classification in 2005 (2), reached by consensus within an international expert symposium that was held in Krickenbeck Castle in Westphalia, Germany (Table 1) (2). Currently, the Krickenbeck classification is the most adopted for its practical clinical use.

The first 24–48 h of a newborn with ARM are fundamental to reaching appropriate information for a correct diagnostic assessment of the malformation and associated anomalies (1). This assessment will help in defining the therapeutic strategy. Screening of associated anomalies is performed with an echocardiogram, abdominal ultrasound, spinal ultrasound, chest and abdominal x-ray, and spinal x-ray. Additionally, the limbs of the patients need adequate clinical evaluation to rule out the presence of orthopedic anomalies that will be further investigated by radiological imaging in the case of any suspicion.

The presence of the ARM itself exposes the patient to the risk of bowel obstruction; therefore, early clarification of the anatomy of the malformation is necessary to decide the surgical strategy: favoring the passage of meconium and stool through the presence of a fistula, treating the malformation primarily, or opening a colostomy (4). To understand the type of ARM, a meticulous examination of the perineum must be performed first. In all patients, particularly in females, this assessment will evaluate orifices (number and location) to look for a fistula (perineal or vestibular). When a visible fistula is evident, calibration of the fistula will help the passage of meconium, thus avoiding urgent surgery. In the absence of a visible fistula, the presence of meconium in the urine should be checked in males, with direct visualization or by urine analysis (4). With a good index of suspicion, the combination of these evaluations will give us enough information to formulate some hypotheses. To better clarify ideas, in the past, an upside-down film in the lateral position, called “invertogram,” was proposed, with the specific purpose of measuring the distance between the anal dimple and the blind end of the rectum (11). To avoid the risk of vomiting and aspiration, an invertogram was then substituted by CTLxR, being able to provide the same information (7).

In the absence of an evident fistula, opening a colostomy always represents a safe choice to efficiently provide the newborn with the possibility of passing stools and to avoid perineal damage due to uncertainty about anatomy during surgical repair. A primary repair can be considered in some patients in referral centers with adequate colorectal expertise (16). In Peña and Levitt's flow chart (3) for the management of male patients with ARM, CTLxR is proposed after 20–24 h of life, and the coccyx is considered as the landmark to decide the strategy: if the rectal gas is below the coccyx, in the absence of associated anomalies, a primary repair can be considered as an alternative to colostomy, while with rectal gas above the coccyx, a colostomy is recommended. The ARM-Net Consortium proposed instead to measure the distance of the lowest level of the rectal pouch and skin in centimeters (either by CTLxR or ultrasound): when the distance is <1 cm (with normal buttocks, normal spine, normal sacrum, and normal urinalysis), a primary repair can be performed; in all other cases, a colostomy is proposed as the treatment of choice (4).

With our study, we aimed to propose using specific anatomical landmarks in the assessment of the CTLxR: the PC line, the I line, and the “pigeon sign”, to better clarify the anatomy and to optimize the information that imaging can provide. The rectum is normally surrounded by funnel-like muscles. The PC line corresponds to the upper limit of this funnel, meaning the level of the levator ani attachment to the pelvic wall. It represents a reference line for the pelvic floor on imaging studies (7) and anatomically corresponds to the level of bladder neck, verumontanum, and pelvic reflection. In ARM, the rectum usually passes through the funnel-like muscles ending ectopically, except for the rectovesical fistulas, which represent the highest of all defects in male patients, where the rectum is not surrounded by this sphincter mechanism, ending directly in the bladder neck (17). The ischiatic line, on the other side, usually corresponds to the superior surface of the bulbar urethra. This is why we decided to consider the possibility of a rectovesical fistula when the rectal pouch is ending above the PC line, prostatic when ending between PC and I lines, and bulbar when at the level/below the I line (17). The rectum is normally surrounded by the striated muscles with a significant tone. These muscles keep the distal part of the rectum collapsed until the intraluminal pressure is high enough to overcome the muscle tone. This physiologically happens usually after 24 h of life, when the bowel gas progresses distally. It is important to perform the CTLxR after at least 24 h of life to obtain a reliable image that does not underestimate the level of the rectal pouch and may eventually disclose the presence of a fistula. The first 24 h can be dedicated to the investigation of associated anomalies.

Other radiologic investigations, such as perineal ultrasound (18, 19), CT scan (20), and magnetic resonance imaging (21), have been proposed to determine the position of the rectum. Our results show that CTLxR, performed after 24 h, and its assessment with the use of PC and I line plus the evaluation of the “pigeon sign”, is useful to hypothesize the diagnosis and start planning the surgery. Additionally, we obtained high specificity, sensitivity, and positive predictive value of the “pigeon sign” in identifying urinary fistula. Therefore, a urinary fistula can be diagnosed in the presence of this sign. A urinary fistula with a negative predictive value of 50% cannot be excluded if the “pigeon sign” is absent. Strict adhesion to the method for performing CTLxR can improve the negative predictive value, although the possibility of very dense meconium ultimately makes the chance of obtaining a value of 100% remote.

Another important reason to optimize the preoperative information that imaging can provide is that the pediatric surgery community is moving toward the primary repair of ARMs (3, 4, 22). Primary repair, in fact, can reduce the number of operations, avoiding the risks of multiple general anesthesia and surgery and reducing the family stress in dealing with multiple operations and managing stoma at home. In this trail, we propose the primary repair of the urinary fistula, defined as the PRUF technique, when we have proof that a male patient with ARM has a urinary fistula (16). Anyway, considering the risks of performing an anorectoplasty at birth and the functional consequences of inappropriate surgery with unsatisfactory results in the neonatal age, we believe that this approach should be performed only by expert pediatric surgeons with skills in colorectal surgery and only after gaining optimal information with clinical examination and imaging. A correct definition of the level of the rectal pouch and the presence of a urinary fistula influence surgical approaches and outcomes.

For this reason, we believe that CTLxR still represents an important diagnostic tool when correctly interpreted, with the help of objective elements such as the PC and I lines and the “pigeon sign”. CTLxR should be performed after 24 h of life and should be of good quality, meaning that a marker should be placed on the anal dimple and the patient must be positioned correctly with the two femurs perfectly aligned, staying in the prone position for at least 3–5 min. Moreover, CTLxR can be obtained quite easily, even in very resource-restrained hospitals and can be interpreted by surgeons directly. With our preliminary study, we would like to provide objective tools to be used by all pediatric surgeons worldwide. It would be interesting to compare the use of x-ray images with the ultrasound images in the facilities where perineal ultrasound evaluation is feasible.

If the three-step approach is chosen, high-pressure distal colostogram is essential to clarify the anatomy before anorectoplasty. High-pressure distal colostogram has been performed for many years, and several papers have been published focusing on its techniques and pitfalls (9, 10, 13). Despite this, the interpretation of colostogram images may be influenced by several factors, such as the quality of the study itself, the collaboration of patients and families, and especially the experience of the radiologist and surgeon. For the same reason previously mentioned, enough intraluminal pressure is necessary when performing this investigation to avoid misdiagnosing the type of ARM. In fact, with low pressures, a urinary fistula could be missed, and the level of the malformation could appear higher than it is.

Recently, Midrio et al. (6), on behalf of the ARM-Net Consortium, reported a poor agreement among experienced pediatric colorectal surgeons on preoperative colostograms of males with ARM. In fact, the agreement between the image-based rating of surgeons and the intraoperative findings ranged from 0.06 to 0.45, and the interobserver variation was very high. In their paper, the authors concluded that techniques and analyses of images need to be improved to perform homogeneous evaluations. The use of the anatomical landmarks suggested by our study, with an almost perfect agreement with the final diagnosis, definitely helps in classifying the ARM preoperatively at high-pressure distal colostogram and providing an objective assessment. The results of our study show perfect intraobserver agreement when colostogram images were evaluated twice by the same operator, and the substantial intraobserver agreement when CTLxR images were evaluated twice by the same operator highlights the great reproducibility and the objectiveness of the PC and I lines and the “pigeon sign”.

With our study, we aim to provide a more objective method to interpret colostograms with the use of PC and I lines to analyzing these images reproducible all over the world. Moreover, we are implementing these lines in evaluating the very first radiological examination given to ARM patients, combining them with the “pigeon sign”. We hope that in the future more centers can join us in the evaluation of CTLxR and colostograms using the PC line, I line, and “pigeon sign” to increase the numbers and validate our methods. We aim to provide better care to ARM patients taking the most possible information from the radiological exams to refine our surgical plans; our final goal for the future is to understand whether we can tailor our surgery directly on CTLxR, saving radiological exposure and offering a safe primary repair even in the case of rectourinary fistulas at tertiary-care colorectal centers.

Our study presents some limitations. The first limit is represented by the small number of patients included in the study. This is because we decided to include only patients who received the three-step approach and who underwent both imaging studies at our center. This choice was made to limit the bias of including images performed at other centers, which may have been acquired potentially with different modalities and techniques. In addition, the high agreement observed in our study might be justified by the fact that our center is a referral center for pediatric colorectal diseases, especially ARMs, and that all the studies were performed by dedicated pediatric surgeons and radiologists.

## Conclusions

Our preliminary results show that objective methods, such as PC and I lines, and “pigeon sign”, are very useful to better clarify the anatomy before surgery. Having objective methods for the evaluation is helpful in making the study reproducible and assessable even to less experienced surgeons and radiologists. The correct and meticulous execution of the radiologic studies from a technical point of view remains the essential prerequisite for their correct interpretation and for offering the best surgical care to the patient.

## Data Availability

The raw data supporting the conclusions of this article will be made available by the authors without undue reservation.
